# Characteristics of Elderly Care Work That Influence Care Workers’ Turnover Intentions

**DOI:** 10.3390/healthcare9030259

**Published:** 2021-03-01

**Authors:** Jeongmi Lim

**Affiliations:** Department of Population Policy Research, Korea Institute for Health and Social Affairs, Sejong Special Self-Governing City 30147, Korea; jekljm@kihasa.re.kr

**Keywords:** care worker, elderly care work, turnover intention, long-term care

## Abstract

Turnover and retention of care workers in long-term care (LTC) settings is an important issue. However, much research about turnover in LTC settings has focused on licensed nurses or nurse assistants. Moreover, many studies have utilized quantitative methods. The purpose of this study was to understand the characteristics of elderly care work that influence the turnover intentions of care workers in LTC. In-depth interviews were conducted with 10 care workers and analyzed using the content analysis method. As a result, seven categories were extracted as the characteristics of the elderly care work associated with turnover, including low social appreciation about care work, precarious employment, unprotected labor rights and safety, an unfair wage system, unclear scope and role of work, absence of training and supervision to enhance professionalism, and emotional labor. For the turnover prevention and retention of care workers, it is necessary to resolve the insecurity of care work. In particular, guidelines for improving the wage level and working conditions of care workers should be instituted, and at the same time, government supervision is required. Education is necessary to strengthen the professionalism of care workers and ensure skilled care work.

## 1. Introduction

With the aging population, elderly care work, which was provided in-home for free or traded in the informal labor market, was institutionalized as social care due to the growing prevalence of long-term care insurance for the aged in 2008. The implementation of long-term care insurance in Korea has expanded the target of the services from the low-income elderly, who were required to be protected, to the general elderly population. It has also led to long-term care staff (e.g., care workers, registered nurses, nursing aides, and social workers) playing a pivotal role in elderly care. Among them, care workers are individuals who provide direct care service to long-term care recipients at home or in LTC facilities. 

The number of care workers reached 360,000 in 2018, accounting for 90% of all long-term care staff, and they are the backbone of the long-term care insurance system [1]. However, since the starting point of elderly care work was unpaid domestic work provided in the home, it is still recognized as work that any woman can carry out despite its conversion to paid work (e.g., home care, residential care) [2,3]. As a result, it is still not properly recognized as a fully-fledged career with its own qualification standards, training requirements, and pay system [4]. This is also related to the fact that lot of care work is still provided within the family. Care work is regarded as female labor, low-wage labor, unskilled labor, and physical labor, regardless of whether it is at-home care or residential care [5,6,7]. The social value of care work has also been devalued [5,6]. As a result, there is a serious level of care workers providing elderly care at home care facilities or residential care facilities leaving from the long-term care market [8]. Moreover, the shortage of manpower continues [8]. 

In the meantime, if one wants to work as a care worker in the South Korean long-term care market, it is mandatory to obtain a care worker qualification. However, only 23.9% of qualification holders are working as care workers [1]. The labor shortage of care workers is serious in the long-term care market [9]. Moreover, the turnover rate of care workers is also very high (the mean turnover rate in 2011 was 41%) [10]. Particularly, since care workers are people who provide services directly to the elderly, their high turnover rate negatively affects the quality of services. Thus, it is necessary to understand the reasons for the turnover of care workers and to support their retention.

Japan, similar to South Korea, provides long-term care services based on social insurance and has experienced a shortage of care workers since 2013, and it is reported that it would be necessary to hire 60,000 new care workers annually [11]. The turnover rate is also on the high side, at 16.7% [12]. As a result, a government-led large-scale survey was conducted to identify the concerns of workers, and it was found that low wages, long working hours, and heavy workload were the main causes of the manpower turnover and retention barriers [11,12]. Consequently, to resolve the issues, the Japanese government institutionalized the support to give better treatment, expenses support for improving the working environment, and support for education costs of attaining qualifications [11]. Likewise, European countries have paid attention to policies for care worker’s turnover prevention and supply in preparation for the increase in the demand for elderly care. The use and training of immigrants, improved skills through education and training, education expense support, building good leadership and communication skills, improvement of treatment, and creating a supportive work environment (e.g., supportive leadership) have been promoted as potential ways to overcome the lack of care workers [8,13,14,15,16]. Specifically, in the data of international research, staff relationships, leadership, and burnout were found to influence nursing staff intentions to leave [17]. Nursing assistant hours per patient day in the nursing home showed the most consistent associations with lower turnover and higher retention [18]. Some studies reported that the development of meaningful relationships with residents and staff, providing adequate incentives, and improving professional learning opportunities are effective for employee turnover prevention and retention [5,16,19]. Other studies reported that less provision of social support by supervisors, colleagues, family, or friends was associated with increased risk of turnover [20]. Higher leadership practice scores were associated with lower nursing staff turnover [15].

In South Korea, a survey reported that it would be necessary to provide an appropriate wage, improve the working environment, and enhance benefits in order to decrease the turnover of care workers [21]. To solve the workforce shortage, it is important to increase the entry of care workers, but it is also important to support them so that they can successfully settle in without leaving their jobs [21]. Other studies reported that it is necessary to increase job satisfaction and alleviate job stress in order to lower employee turnover, as job stress decreases the job satisfaction of care workers and low job satisfaction increases the urge to change jobs [22,23,24]. Several studies found the following as being relevant to care staff turnover: role conflicts, workload, wage payment system, and stress with coworker relationships [10]; and organizational commitment and job satisfaction [25].

However, despite these results, most of the studies conducted in South Korea have focused on analyzing the working conditions of care workers. Only a few studies have examined the factors affecting the turnover intention of care workers in the LTC market. In addition, most of the research on the turnover intention of care staff in LTC settings has utilized quantitative methods [10,22,23,25]. Existing studies have not yet provided a rich and detailed explanation of the reasons for the turnover of care workers. Furthermore, much international research [15,16,17] focused on turnover in LTC settings has focused on nurses. Therefore, factors related to turnover need to be examined in more detail based on the opinions and experiences of care workers.

The purpose of this study was to understand the characteristics of elderly care work that affect the turnover intentions of care workers in LTC, and to suggest measures to prevent care worker turnover and encourage their retention.

### Long-Term Care Insurance (LTCI) System in South Korea

In April 2007, the Elderly Long-Term Care Insurance Law was passed by the National Assembly, and the LTCI has been implemented since July 2008 by the National Health Insurance Service (NHIS) [26]. It resulted from the combination of several social phenomena: changes in population structure, an increase in women’s labor participation, and the change in family structure [27]. LTCI adopted contribution-based social insurance financing [28]. It is basically based on the social insurance but is also financed by the government subsidies and co-payments by the actual beneficiaries of the LTCI [29]. Under this system, all citizens must pay a monthly insurance premium, which is 10.25% of the national health insurance premium, as a LTCI subscriber [30]. Among them, people who have difficulty with activities of daily living for a period of at least 6 months are considered eligible for LTCI, including both those who are 65 years old or older or who are less than 65 years old but have geriatric diseases [31].

Long-term care insurance in Korea has not been properly regulated for long-term care institutions and service workers since the early days of the system’s introduction, so the work environment and labor rights of the care workers have not been properly guaranteed. Due to inadequate government control and supervision of long-term care institutions and service workers, labor contracts under the Labor Standards Act are often not applied [21]. In spite of the public resources being invested, there are no guidelines for wages, so wages are formulated through deals between facilities and workers. In response, in order to improve the treatment of care workers, standards were established that mandated personnel expenditures for care staff in the long-term care insurance benefits received by the long-term care institution. However, care workers are still paid less than service workers in similar industries [32].

## 2. Materials and Methods 

### 2.1. Study Participants

The study participants were recruited through the Support Center for Seoul Care Workers in Korea. This center is a public institution run by Seoul city that supports care workers’ counseling (e.g., task, job search, and job change counseling), education, and training. Since many care workers can easily use the center, we decided to request the center to introduce us to interviewees who fit the research focus of this study. We explained the purpose of the survey and the selecting criteria for interview participants to the center’s staff over the phone and asked them to recruit candidates. When selecting the participants, the operating organizations of the facility and type of the facility were considered, and selective sampling was used to include care workers who understood the characteristics of the elderly care work and could describe in detail information on the turnover intention of care workers. Criteria for selecting participants were as follows: (1) participants had at least three years of work experience in long-term care facilities; (2) participants also had thought about leaving the job or had experienced turnover in the field. Ten participants were interviewed. Five participants (i.e., Participants 1, 2, 3, 4, 5) worked for residential care facilities, and the other five participants (i.e., Participants 6, 7, 8, 9, 10) worked for home care services. The characteristics of the participants are presented in Table 1. All participants were women, and the mean age was 59. It has been reported that about 90% of the care workers in Korea are in their 50s or older, and among them, about 40% are in their 60s [32]. Therefore, the age of these interview participants is considered to be representative of care workers nationwide [32].

### 2.2. Data Collection

Group interviews were conducted using a set of semi-structured open-ended questions. We adopted group interviews to facilitate a lively exchange of ideas among the participants. Interviews were conducted from June to September 2019, and each interview was one hour and thirty minutes long. The questions were “What is care work?”, “What makes it difficult to work as a care worker?”, and “What circumstances influence you to leave the workplace?” The interviews were carried out by grouping two or three participants in a conference room located near the facility. Each participant had two or three interviews. This process was carried out until no more new information was forthcoming, and this ended when the same information was repeated. All interviews were recorded using a voice recorder. 

### 2.3. Analysis

The researchers decided to analyze the data from care workers at home care facilities and care workers in residential care facilities as a combined data set because of the small sample size and because care workers in each setting had similar roles. Data was analyzed based on the conventional content analysis method [33]. Following the content analysis guide [33], after the text about the participants’ job-leaving intentions was extracted and compiled, meaning units, codes, subcategories, and categories were created. First, the interview was recorded with prior consent, and the recording was transcribed verbatim. It was reviewed by comparing it to field notes recorded by the researchers to ensure the accuracy of the data. Second, the two researchers underlined meaningful statements or concepts about work and turnover intention as a care worker. They reviewed them repeatedly to ensure an accurate analysis. As this process continued, researchers extracted labels for codes. Then, they created subcategories based on how different codes were related and linked. Similar subcategories were merged into categories through the two researchers’ views. All the researchers expressed consensus on the categories and subcategories.

### 2.4. Rigor and Ethical Considerations

Three researchers, including one field expert with experience in qualitative research, repeatedly reviewed and analyzed the data to assure the rigor for the study. The researchers discussed the results and reached a consensus. The study was deliberated and approved by the Institutional Review Board (No. 2019-30). Interviews were conducted only for care workers who agreed to participate in the study after understanding the objectives and importance of the study via phone or mail in advance. It was explained that they could quit at any time during the interview and that all contents would be anonymous and confidential. The study was conducted after receiving consent from all participants.

## 3. Results

Seven categories were extracted for the factors (e.g., characteristics of elderly care work) that influence turnover intentions of care workers (Table 2). Moreover, various characteristics of elderly care work were connected and intertwined, affecting the turnover of care workers.

### 3.1. Low Social Appreciation about Care Work

#### 3.1.1. Society’s Prejudice against Care Work 

Since there are no age or education restrictions and it was a housework-oriented job, the job attracted middle-aged women in each setting (both at home and at residential facilities). Participant 5 did not have a suitable job when she got older and learned about the care worker career through a recommendation of an acquaintance (residential care facility, RCF, Participant 5). She stated that it was somewhat demanding because of physical labor, but it was not difficult to get the job as long as one tried, as it was similar to housework. 

Such low entry barriers have caused the elderly who use these services perceive that care work is something that any woman can do. In reality, a care worker has been often treated as a housekeeper and evaluated as a person who does unimportant and tough tasks: “An elderly person asked me. Why do you do this job? You’d rather be dead than do this.” (home care facility, HCF, Participant 6). Participants told us that it has made people hesitate to work as care workers and has prompted care workers to leave the sector. Especially for home care, the care work, which is too concentrated on housework, has caused care workers to be considered as housekeepers for users and their families.

The social awareness is so low that care workers cannot say that they are care workers to other people. People say that the job hurts their nerves too much to have that job. Therefore, people just get the qualification, but don’t actually take the job. (HCF, Participant 6)

Someone even changes jobs after obtaining qualifications as social workers or nursing assistants due to the poor perception of care workers. (HCF, Participant 1)

A friend of mine asked me why I was doing such things (e.g., dishwashing, house cleaning, and bathing) while I had a college degree. Whenever it happens, I have considered quitting or leaving the job several times. (HCF, Participant 7)

#### 3.1.2. Lowest Social Status 

Further, the head of institutions of home care and residential care facilities that employ care workers consider care workers as easily replaceable manpower. Participants reported the head of facilities responding to the care workers who expressed difficulties arising from the relationship with the service users that they should quit if they could not handle those difficulties. Participants also explained that the perception and response of the employer makes it hard for care workers to continue working and hinders the entry of new workforce members into the long-term care market. In addition, there was an opinion that care workers were ignored more because the status of care workers was lower than that of nurses’ assistants and it would be better to work as nurses’ aides even when conducting the same tasks (RCF, Participant 5). The low professional status and treatment undermine the professional identity of care workers and encourage turnover.

It is basically “take it or leave it”. We are not treated respectfully. The young will not work when they are treated like this these days. We endure it because we are old. (HCF, Participant 7)

A care worker is in a lower position than a nurses’ aid. It is considered as something anyone can do. Even if we do something similar, the nurses’ assistant is acknowledged more. Their pay is higher as well. I want to change my job after getting a nurses’ assistant qualification. I would like to move to a facility that treats me a little better, even if it is the same care worker. (RCF, Participant 5)

### 3.2. Precarious Employment 

#### Various Forms of Involuntary Unemployment and Turnover 

Care workers at home care facilities have lost their jobs and means of living without any reason due to the death or request of the elderly (e.g., termination of the contract). Due to the loss of work after the death of a service user, Participant 6 had to move to another center because the facility did not quickly connect her to another elderly person. Participant 7 stated that sometimes, the elderly sent a message for the care worker not to come on that day without prior notice, but there was no compensation standard for this. Many participants reported that when it happens, the head of the facility, who is the employer, does not arbitrate between the elderly and the care worker. They also stated that when the user and the employer terminate the contract, they could suddenly become unemployed. The precarious employment, involuntary unemployment, and turnover threaten the livelihood of the care worker and make it difficult for the care worker to work continuously.

If an elderly person suddenly passes away, I lose a job. If we lose a job without knowing when our next assignment will be, we will not have income. I have no choice but to move on to another institution. (HCF, Participant 6)

Sometimes, when I visit the elderly’s house to offer services, there is no answer to the doorbell. If I call the elderly, the person says I am at my daughter’s home. Then, I’ll lose my income for that day. There is no compensation, even if an elderly cancels the service unexpectedly. (HCF, Participant 7)

When I am on my way to work, I sometimes receive a message without any prior notice. It says “Don’t come to work from today.” I am fired because the user does not like me and I feel frustrated. If the user says he or she doesn’t like the service, the head of the facility can’t do anything. Because there are too many home care facilities and competition is severe, nobody wants to lose a user. (HCF, Participant 8) (HCF, Participant 10)

The above case study illustrates that the employment of care workers in residential care facilities is more stable than that of those in home care facilities. However, they also can lose a job or have to move to a new job due to an organization’s closure or sudden business suspension. Various forms of involuntary unemployment, resignation, and turnover occur in each setting. Participants stated that such precarious employment can negatively affect the supply of care workers by obstructing the retention of care workers into the long-term care market and promoting turnover.

The facility manager suddenly closes the facility. Then, I have no choice but to leave a job or move on to another facility. Who would want to work while a job is insecure like that? It is always “take it or leave it.” Young people cannot enter this instable employment situation. (RCF, Participant 1)

### 3.3. Unprotected Labor Rights and Safety

#### 3.3.1. Difficulty in Using Vacation 

Care workers were not able to take vacation freely. Home care workers reported that taking a vacation sometimes led to unemployment and an unavoidable turnover. Some participants stated that even if a substitute worker was dispatched, he or she lost a job because the user did not accept the arrangement. Moreover, the work schedule of the care worker had been adjusted one-on-one with the elderly (or family members) rather than through the facility:

Our work schedule is changed by talking directly with the elderly people and then we report that to the facility. The head of the facility also told me to discuss the use of vacation with the elderly because the elderly hated other workers coming. (HCF, Participant 10)

Therefore, the employees have a hard time because they do not feel like they are protected by their facilities. The lack of service users’ understanding of the labor rights and the poor human resources management of the facilities burden care workers even more.

I want to take leave on the weekend. However, when I take a leave on the weekend, the elderly person hates it. Then, the elderly person asks to replace me with someone else. These are power harassment and oppression. No one protects us. I’ve also even left jobs because of this. (HCF, Participant 6)

In our case, although our facility has a pool of substitute workers, it is hard to take leave because the elderly do not like to be cared for by a substitute worker. Sometimes, I am replaced with another person after taking a vacation. (HCF, Participant 10)

On the other hand, the manager of a nursing home did not recommend care workers to take a vacation because of it being cumbersome to find a substitute. Participant 5 stated that she became tired of continuously working due to an increased work burden and searched for a new job whenever she had a chance.

We are told not to take a vacation or do not get paid for the time. We should be able to work and take a rest just like everyone else. (RCF, Participant 2)

#### 3.3.2. Non-Guaranteed Break Time 

Home care workers can separate working hours and break time. However, care workers in residential care facilities have a hard time using the break time as prescribed in the Labor Standards Act. Since the facilities operate 24 h a day, it is difficult to distinguish between work and rest (Participant 5). Participant 4 stated that it is particularly hard to take a break on the night shift because two care workers must take care of 30 elderly people. All care workers in residential care facilities feel tired because they are always anxious about what may happen and they cannot rest properly even if they can sleep at night. Since they work continuously without resting well during breaks, care workers suffer from more job stress. Job stress lowers the job satisfaction of care workers and increases the turnover rate [22].

The manager tries to pay the allowance for night work less and reduce work hours. It doesn’t mean that we can take a rest during a break. We have to look after the elderly because we don’t know what may happen to them. It seems that you don’t have to abide by the Labor Standards Act. Thinking about this makes me more stressed. (RCF, Participant 1)

Two care workers take care of 30 people at night and it does not feel like resting even if we lie down on the sofa in the office to rest. I get tired more easily because there are more things to do at night. (RCF, Participant 4)

#### 3.3.3. Poor Human Resources Management Functions of Institutions and Transferred Responsibilities 

A care worker was told by a nursing facility that they should directly hire and pay a substitute worker. This suggests that the human resources management functions of the facility was non-existent. It was also pointed out that care workers who hired a substitute worker had to be responsible for any accidents caused by the substitute worker, and this burdened care workers even more. Although care workers are forced by the facility to be responsible for many things, their labor rights, which they deserve as workers, are not protected. Participant 2 and Participant 3 stated that the large workload made them consider leaving their jobs. Unclear responsibilities in human resources management can exacerbate the turnover of care workers, because it leads to the exploitation of care workers’ labor rights, further threatening the safety of users and deteriorating the quality of services.

We are like self-employed people. What company’s employee directly hires a substitute worker and pays wages? If an elderly person falls and gets hurt due to the mistake of a substitute, we are responsible for that. It is a big burden. (RCF, Participant 2)

#### 3.3.4. Working Environment Vulnerable to Sexual Harassment 

It was said that a care worker experienced sexual harassment when the worker worked at an elderly male’s home. Some employees eventually left the job because the situation was not improved even after consulting with a manager. Neither the facility nor the government protects the safety of care workers.

I think sexual harassment is a disease. It does not get better even after consulting. Many care workers changed their careers because of it. I’ve talked to the manager but the attitude of the user has not changed. I’ve been insisting to the government all along that we have to work in pairs … (HCF, Participant 7)

#### 3.3.5. Work-Related Illness and Resignation Request 

Care workers who work in residential care facilities often cannot receive medical treatment even if they are sick due to the care work because they were not able to visit the doctor’s office during work hours. Participant 1 stated: “My waist and knee hurt more as time passed because I did not receive appropriate medical treatment. I want to quit working because I am not sure how much longer I can work. If I could find a facility that cares about the health of its staff, I would leave this job immediately.” Care workers are often exposed to infectious diseases such as influenza or tuberculosis because they take care of the elderly who are vulnerable to such diseases. In that case, they are often asked to leave and receive unemployment benefits even if they receive medical expenses from the facility. Although the facility’s manager says that they would secure another position for the sick care worker, it is difficult to continuously hire a non-working care worker.

If an employee is injured while working, the facility has to compensate and treat you medically. Many people have quit working because there is no such benefit. We can’t receive medical treatment during working hours. I would like to quit and take a rest, but I can’t quit because I have to work for a living. (RCF, Participant 2)

An elder became ill from tuberculosis. I also received medical treatment for about 5 months because of tuberculosis. I was told I could return, but I could not because the manager was disgusted. I left the company for unemployment benefits while receiving treatment. (RCF, Participant 5)

I got urticaria because of an old man. It was difficult for me to continue working because it was contagious. (RCF, Participant 1)

### 3.4. Unfair Wage System 

#### 3.4.1. Low Wages (No Wage Guidelines) 

Since care workers receive low wages despite the high intensity of work, including long working hours and three work shifts, many care workers resign or move to other higher-paying facilities. Particularly, in the study, Participant 1 and Participant 5 complained that their actual wages were not raised even when the minimum wage was increased because the manager extended the night break time. Participant 2 indicated that they received low wages because the government did not provide wage guidelines. Since long-term care is operated by social insurance, the government has the responsibility to supervise the overall operation of the system and facilities. However, each facility had a different wage system, and some provided low wages and poor treatment because the government did not supervise the facility properly. Some participants reported that many new employees quit after working only a few days because the wages were too low compared to other hard jobs, especially for care workers in residential facilities. Care workers who have worked for several years try to obtain different jobs which pay slightly higher. The idea that they are not paid fairly in relation to the high intensity of their labor increases the turnover intention of existing employees.

I can’t believe the wages are so low for even working at night. Many people quit just after working for a few days. If you don’t get paid for all the work you’ve done, rumors will soon start to circulate. (HCF, Participant 1)

It would be nice if the wages or bonuses were similar between facilities. Since the government does not give a payscale guideline or supervise the facilities, people want to move to a different facility to get paid a little better. (RCF, Participant 2)

#### 3.4.2. Wage System That Does Not Take into Account Previous Experience 

Skilled care workers are important in improving the quality of care services. This is because the experience and skills of a care worker are required to perform suitable care depending on the user’s needs and conditions [3]. However, the experience and skills of a care worker in residential and home care were not reflected in the wage of the worker in practice. A system that does not acknowledge skilled workers encourages the turnover of human resources. Accordingly, the government recently introduced a “long-term service allowance” to improve the service quality and to support turnover prevention and the retention of care workers. However, it is still necessary to extend the purpose and coverage of the long-term service incentives. It is because many care workers at home care facilities are unemployed involuntarily and then change jobs. Participant 5 stated: “I have worked for over 10 years as a care worker, but if I quit working and return to work, I will receive the same treatment as a new employee. My previous experience will be gone. I am also not entitled to a long-term service allowance” When they move to another facility, their previous experience will not be recognized and they will not be able to receive the long-term service allowances. Even if one has worked as a care worker for a long time, previous experience is not acknowledged and the person receives lower wages. Even if an experienced care worker receives the allowance, there is little difference in the wages between experienced and novice care workers. Since the long-term care insurance system pays care insurance compensation according to the hours of service provided, the experience cannot be reflected in wages in the current structure.

Even if I am promoted to a team leader position, it has nothing to do with my salary. The salary remains the same regardless of my work experience. There is no hope. (RCF, Participant 1)

We get paid minimum wage, even if we have a longer career. I would leave jobs if I could find a facility with a slightly higher wage. (HCF, Participant 6)

The elderly person (eligible for home care services) died and I had no choice but to move to another home care facility. In this case, we are not entitled to long-term service allowances. Even if the system is in place, we are not covered. (HCF, Participant 10)

### 3.5. Unclear Scope and Role of Work

#### 3.5.1. Unfair Work Demands 

Since the task of a care worker in home care is not clearly defined, care workers often have to do tasks other than those listed in the care plan. This ambiguity means the care worker often performs tasks as directed by the user or the user’s family. In fact, almost all care workers in home care facilities did laundry or cleaned the house and performed tasks other than supporting the user, and it was difficult to refuse these requests; examples included washing the family’s clothes, bathing puppies, and soaking kimchi (HCF, Participants 6, 7, 8).

If the care worker turned down the request of the family, the family asked to replace the care worker. A participant said that the facility took the side of the user for these unfair requests instead of protecting the employees. Care workers who cannot accommodate these demands will inevitably have to move to another facility. The scope of care work depended on the relationship with the user [3], and this was a causal factor that exhausted care workers. Poorly acknowledged care skills combined with an uncertain scope of work and unfair work demands hindered the retention of care workers and affected their turnover.

Users are making unreasonable demands. I was once asked to clean the windows. I said that I would do it now, but it was not my job. I guess that the user was a little offended. Then, I received a text message saying if the work was too hard for me, I could stop working. It’s so unfair and I felt useless. The head of the facility also just cares about the user’s perspective. She doesn’t help us. (HCF, Participant 7)

#### 3.5.2. Heavy Workload 

The uncertainty of the work scope was equally applied to care workers in residential care facilities, leading to overwork and burnout. Care workers in residential care facilities do not have defined work priorities and are always busy, so they are exhausted by the time they leave work. They do not have time to care for the user’s mind. Because they were too busy every day, they felt like taking training courses for improving skills, such as dementia training, was a total waste of time. The ongoing hard work was increasing the turnover intentions of care workers.

I have to clean, do the laundry, or throw away the trash. It hurts my back. I do not have work priorities. I get tired after doing it alone. I wish I could give care like talking kindly with the elderly. I would like to leave jobs if such care is available. (RCF, Participant 1)

I have too much to do in addition to caring for the elderly. We have to do miscellaneous work and complete dementia training and humanistic education every month, but I just want to take a rest. I feel like taking courses such as dementia training is a total waste of time. (RCF, Participant 5)

### 3.6. Absence of Training and Supervision to Enhance Professionalism

#### 3.6.1. Poor Understanding of Work 

A poor understanding of the work hindered the entry of care workers into the labor market and encouraged them to change jobs in the early stages. Although it seems that people understand the nature of the job to a certain extent, many care workers quit working as soon as they start working because of the gap between expectation and reality. Care workers quit working or moved to different jobs because their expectations and the reality were different, or the job did not meet their aptitudes.

Many people quit working without an exact reason after seeing the actual care services because their self-esteem was hurt. (HCF, Participant 7)

Even if I get a qualification, the field is very different from the knowledge given in the book. People said they were afraid of working as a care worker. They quit by saying that they would do it later. Many people leave their jobs after a few months of work.” (RCF, Participant 4)

#### 3.6.2. Better Understanding of the Work Is Needed for Better Care 

Above all, training is needed to relieve the vague anxiety and difficulties in caring for the elderly with dementia. The anxiety due to the lack of understanding about dementia while conducting work increases the workload of employees and encourages turnover [34].

An elderly person may show unintelligible behavior suddenly, examine me to see if I steal anything, or use foul language towards me. If you don’t understand dementia, you can’t work getting paid this low. (RCF, Participant 5)

#### 3.6.3. Insufficient Training Opportunities 

Care workers do not have sufficient training and education opportunities to improve their skills after beginning employment. They rarely have an opportunity to receiving counseling training to care for the users’ mind and body, fall-prevention training, or training for understanding and coping with the users’ behaviors. It is worse for small facilities operated by an individual [35]. Care workers have to invest personal time and expense to receive training but attending training does not increase their wages.

A lot of people say that they don’t understand why they should take courses while they are too busy. (HCF, Participant 8)

We have few opportunities for the education itself. We need to be educated and enhance self-development, but we can’t do that, so we get tired of our tasks quickly. So if there is a facility that can pay us more, we will easily consider leaving the job. (HCF, Participant 6)

I would like to receive some training from experts in advance, such as how to deal with mentally troubled people. (RCF, Participant 5)

#### 3.6.4. Insecurity Due to the Absence of Supervision 

Some participants reported that they feel difficulty and unease when performing tasks because there is no adequate feedback or supervision. Care workers in home care facilities require a higher-level skillset because they work alone in the user’s home, unlike care workers in residential care facilities who can get help from a nurse or team leader in case of an emergency [36]. Participant 6 stated, “If there is a supervisor who can help me to overcome difficulty, tasks can become easier and it is helpful for self-development. However, there is no supervisor in reality and I am concerned about it. I would like to leave my job”.

I am sometimes worried and anxious about whether I am doing the right thing for the elderly. Is this care correct? In particular, I am afraid that I may encounter an accident or an emergency situation. (RCF, Participant 5)

At my previous facility, I had to work alone at night-time, which made me feel uneasy, so I moved to this facility. (RCF, Participant 1)

If there is a case meeting once a week, you can get help when it’s difficult to give proper care. I bet that there are more places not offering it. I am working for an old institution, so it’s fun to have a meeting. I can even present my own opinion … I also got my nurse’s aide certificate. I want to work here continuously” (HCF, Participant 9)

### 3.7. Emotional Labor

#### Emotional Control and Mental Stress 

Care workers reported that they experience severe stress and pressure because they have to control their emotions while performing tasks and provide services with an understanding the user’s needs and circumstances as much as possible. Participants stated that stress is further maximized when the user dies. The problem is that the care worker has to endure it entirely alone. Mental stress and post-traumatic disorder (PTSD) impede care workers working continuously because they experience vague anxiety and fear.

It’s emotional labor. Even if you feel bad, you always have to smile as if you feel good. (HCF, Participant 8)

Although I have worked for a long time, sometimes when an elderly person passes away, it really bothers me for a while because the memory stays with me. I even quit working because I was scared of thinking what if all the elderly die at once when I worked alone at night. (RCF, Participant 2)

In addition to conflict with users, some people left the job when they had conflicts with co-workers or supervisors because they had nobody to confide in or consult with. The condition gives them more concerns and it also makes it difficult to adapt.

I have to work together for 24 h. If we are not like-minded, it’s very hard. It would be nice to have a co-worker whom you can freely talk to about issues. If we feel inconvenienced with a colleague, we often quit working and move to another facility. (RCF, Participant 2)

## 4. Discussion

This study aimed to analyze the characteristics of care work perceived by care workers in long-term care facilities and examine how these characteristics influenced the turnover intentions of care workers. In other words, the purpose of this study was to examine the factors associated with the turnover of care workers in LTC settings and to propose strategies to prevent turnover and increase retention. 

The results of this study showed that the factors (e.g., characteristics of elderly care work) that influence turnover perceived by care workers were low social appreciation for care work, precarious employment, unprotected labor rights and safety, an unfair wage system, unclear scope and role of work, absence of training and supervision to enhance professionalism, and emotional labor. Moreover, various factors were connected and intertwined, affecting the turnover of care workers. Similarities and differences were found in the factors influencing turnover by care workers working in homes and in residential facilities. Low social appreciation, an unfair wage system, unclear scope and role of work, absence of training, and emotional labor were common difficulties experienced by all care workers regardless of the type of setting (home or residential facility) and also influenced turnover. In contrast, precarious employment and unprotected labor rights and safety had different levels of effects on the turnover intentions of residential facility care workers and home care workers. For example, since residential facility care workers need to provide care around the clock, it is difficult to distinguish work hours and rest hours, forcing them to work long hours, which in turn encourages them to leave their jobs. On the other hand, home care workers are paid on an hourly basis and have no concept of rest hours during work. Therefore, this does not affect turnover. Employment instability was experienced more frequently by home care workers than by residential facility care workers. In other words, employment instability had a greater impact on home care workers than on residential facility care workers.

First of all, the low entry barrier in the absence of age or education restrictions and the housework-oriented work mean care workers receive a poor social consideration. The low social consideration of care work was also promoting the turnover of care workers. These results are similar to those of previous studies [5,6]. However, in addition to the existing research results, the results of this study explain the turnover of care workers under the unique institutional conditions of Korea. For example, the low social reputation caused by a lack of government supervision and manpower management functions led not only the users but also the heads of the facilities to treat the care workers as easily replaceable. Consequently, the managers responded to distress arising from the relationships in a way that showed that care workers should absorb it or stop working. This was one factor that made workers leave their jobs. Thus, it is necessary to eliminate the low social status and negative perception of elderly care workers through systematized training, thereby strengthening the professionalism of care workers. This is because the social approval of the value of care work can only be secured through education and training [6]. A trained care worker can provide quality services just by observing the facial expressions of the elderly or the behavior of the elderly with dementia because they can identify unexpressed needs [37,38,39,40]. They also can prevent the diseases from getting worse by cooking according to the elderly’s dietary needs or by identifying the health status of the elderly from their urine. Improving the professionalism by systematizing the training appropriate to the career level of care workers can help home care service users and their families to recognize that care work requires specialized knowledge and skills and is not something that anyone can do. Moreover, it can increase the value of care work and improve the social standing of formal care work [6]. 

Secondly, the absence of training and supervision to enhance professionalism and decrease emotional stress affected the turnover of care workers. These results were similar to those of previous studies showing that the absence of training opportunities aggravated the lack of professionalism of care workers [35] and encouraged turnover [15]. Quality of care requires staff understanding and autonomy in their work [16,37]. Above all, training is needed to relieve the vague anxiety and difficulties in caring for the elderly with dementia [37,40]. However, care workers do not have enough opportunities to improve their skills through training after recruitment, and receiving training on vacation is not compensated. This prevents care workers from becoming skilled workers. When a service user passes away, the mental stress of the care worker is greatly amplified. In response, the absence of supervision increases anxiety and fear, thereby increasing job turnover. 

Therefore, improvement in the quality of training must be affected by restructuring the system of care worker education. For example, there is a need for standardized educational content and practice-centered education, and education related to dementia, rehabilitation, and care skills. Several studies reported that providing learning opportunities and professional career development help improve staff retention [15,41]. Supervision for helping care workers understand the death of users in the facility and peer counseling will alleviate the mental and physical fatigue and stress experienced by care workers. 

Thirdly, care workers at home care encountered diverse situations of involuntary unemployment and turnover. Furthermore, this employment instability, combined with income instability, labor rights violations, safety instability, and unclear scope of work, weakened the will of care workers to provide continuous service. Care workers in residential care facilities worked overtime because their breaks were not adequately provided. Moreover, home care workers often received unfair requests from users because the scope of their work was not clearly defined as compared to care workers in a residential care facility. Care workers in home care facilities tend to be exposed to sexual harassment more frequently because they provide one-on-one care to users. However, neither the employer nor the government tries to protect them. The unique multidimensional instability factors of care work are interlinked, and they cumulatively influence the turnover of the care worker to other LTC facilities. These are similar to previous findings indicating that low wages, unclear work scope, and excessive workload led to a shortage of manpower in the long-term care market [38,39].

Therefore, institutional support is needed to resolve the multidimensional instability of care work (e.g., employment instability, income instability, labor rights and safety instability, and unclear scope of work). It is urgent to have a labor-management manual on the operation of long-term care facilities in order to stabilize various forms of involuntary unemployment and to guarantee workers’ labor rights. At the same time, government supervision is required. This, like the above suggestions, needs to be applied to both residential and home care facility care workers. In the case of care workers at home care facilities, employment instability was higher than that of care workers in residential care facilities, as well as the number of unreasonable work demands from users and families, which encouraged turnover; whereas, in the case of care workers in residential care facilities, the difficulties of working long hours and not being able to use break times were the biggest factors in turnover. In light of these facts, it is necessary to create a labor-management manual. Specifically, it is necessary to make a manual on matters related to highly controversial labor conditions such as working conditions, wage levels, method of providing pay in the case of the service cancellation of the user, and resting in accordance with the Labor Standards Act. Particularly, a lot of small facilities in South Korea are operated by individuals, and employers cannot hire or consult a certified labor attorney. Therefore, it will be necessary for the Health Insurance Corporation, the insurer, to advise and supervise the proper labor and employment management of the employer by deploying a labor inspector to the facility. 

Above all, it is necessary to improve the unfair wage system. The government, which operates the long-term care insurance system, sets a low insurance fee while insisting on low cost and high efficiency and hires care workers indirectly through long-term care facilities. To make it worse, the wage scale and working conditions of care workers were lowered because they were determined by free-market principles among long-term care facilities under the low insurance fee condition [39]. In order to increase the value of elderly care work and prevent worker turnover, there needs to be a revision in the wage system based on training. For example, the current housekeeping-centered care tasks need to be developed into more specialized tasks based on training, such as nutrition management, bedsore management, and dementia care for the elderly. When care workers acquire advanced qualifications (e.g., superior care worker, care manager, etc.), as is the case in Japan, compensation needs to be reorganized so that it is reflected in their wages. In addition, there is currently no official promotion system of the long-term care market in Korea. Consequently, there is no wage difference between new employees and experienced employees, which can impede work and organizational commitment [24]. It is urgent to restructure the current wage system and introduce a promotion system that compensates for experience and skills (qualification acquisition) to facilitate the entry of new workers and promote the retention of existing workers. 

The original points of this study are as follows: First, this study sought to provide detailed information on the characteristics of Korea’s unique context of elderly care work and the turnover intention of care workers. For example, the devaluation of care work is similar in both Japan and Korea [3,5,6,24], but in the case of Japanese care workers, it is difficult to see unstable employment or difficulty in using vacation as factors that encourage the turnover of care workers [7,42]. The main factors driving the turnover of care workers of Korea can be seen as a result of the combination of the institutional situation (lack of manpower management functions, lack of government supervision, and underfunded LTC system) and the cultural situation (low esteem of care workers). For this reason, it is necessary to understand Korea’s unique situation in order to suggest a more effective turnover-prevention plan. Second, previous studies have analyzed the factors of turnover from either the perspective of workers at residential care facilities or those at home care facilities [3,25], but this study was able to examine the reasons of turnover comprehensively from both perspectives. Although all care workers working in home settings and in residential settings suffer from problems, such as low wages and employment insecurity, this study was able to understand how the differences in care settings created their own unique set of characteristics (low wages, employment instability, working hours, etc.). This study will hopefully provide policymakers with specific directions for preventing turnover and stimulating retention. 

This study has some limitations. First, since the qualitative study was conducted with a relatively small number of participants, expanded research is required in order to verify and further generalize the results of this study. Second, this study has limitations in examining what characteristics of care work should be intervened in more intensively for preventing the turnover of care workers and encouraging stable retention. Therefore, it is required to review the factors that should be intervened in preferentially for preventing turnover and increasing retention of care workers through future surveys. Third, additional study is needed on why qualified care workers are not entering the long-term care market in order to increase retention. Finally, this was a qualitative study conducted prior to the COVID-19 pandemic. Therefore, it was not possible to include information on how employment and income instability among care workers deepened during the pandemic, regarding changes in work intensity and stress about infections. Additional research is needed on the characteristics of elderly care work and turnover since the pandemic.

## 5. Conclusions 

Various characteristics of elderly care work are connected and intertwined, affecting the turnover of care workers. The low social evaluation of care work, combined with employment instability, an unfair wage system, and unclear scope and role of the job (unfair work demands), encourage care workers to leave their jobs. It is essential to resolve the instability of care work to prevent the turnover of care workers and encourage their retention. The government is required to supervise care facilities actively while providing guidelines for improving wage levels and working conditions. It will be also necessary to systematically improve the skill of care workers through the development of educational programs that can strengthen professionalism.

## Figures and Tables

**Table 1:** Characteristics of participants.

Number	Operating Organizations	Age	Work Experience	Experience Leaving a Job
1	Privately operated facilities	64	11 years	Yes (3 times)
2	Social welfare corporation	65	6 years	Yes (2 times)
3	Social welfare corporation	61	9 years	Yes (1 time)
4	Social welfare corporation	55	7 years	No
5	Privately operated facilities	60	5 years	Yes (1 times)
6	Cooperative association	68	11 years	Yes (2 times)
7	Social welfare corporation	61	11 years	Yes (3 times)
8	Privately operated facilities	55	10 years	Yes (2 times)
9	Religious corporation	60	10 years	No
10	Social welfare corporation	43	3 years	Yes (1 times)

**Table 2:** Characteristics of elderly care work that influence turnover intentions of care workers.

Main Categories	Subcategories
Low social appreciation about care work	Society’s prejudice against care work Lowest social status
Precarious employment	Various forms of involuntary unemployment and turnover
Unprotected labor rights and safety	Difficulty in using vacation Non-guaranteed break time Poor human resources management of facilities and transferred responsibilities Work environment vulnerable to sexual harassment Work-related illnesses and resignation request
Unfair wage system	Low wages (no wage guideline) Wage system that does not take into account previous experience
Unclear scope and role of work	Unfair work demands Heavy workload
Absence of training and supervision to enhance professionalism	Poor understanding of work Insufficient training opportunities Insecurity due to the absence of supervision
Emotional labor	Emotional control issues and mental stress

## Data Availability

The data presented in this study are available on reasonable request from the author.
